# Management of relapsed ovarian cancer: a review

**DOI:** 10.1186/s40064-016-2660-0

**Published:** 2016-07-28

**Authors:** Gonzalo H. Giornelli

**Affiliations:** Genital-Urinary Department, Instituto Alexander Fleming, Cramer 1180, C1426ANZ Buenos Aires, Argentina

**Keywords:** Ovarian cancer, Cytoreductive surgery, Chemotherapy, Relapse

## Abstract

Around 70 % of ovarian cancer patients relapse after primary cytoreductive surgery and standard first-line chemotherapy. The biology of relapse remains unclear, but cancer stem cells seem to play an important role. There are still some areas of controversy on how to manage these relapses and or progressions that occur almost unavoidably in the course of this disease with shorter intervals between them as the natural history of this disease develops. The goal of treatments investigated in this neoplasm has shifted to maintenance therapy, trying to extend the progression free intervals in a disease that is becoming more and more protracted.

## Background

**Fig. 1:**
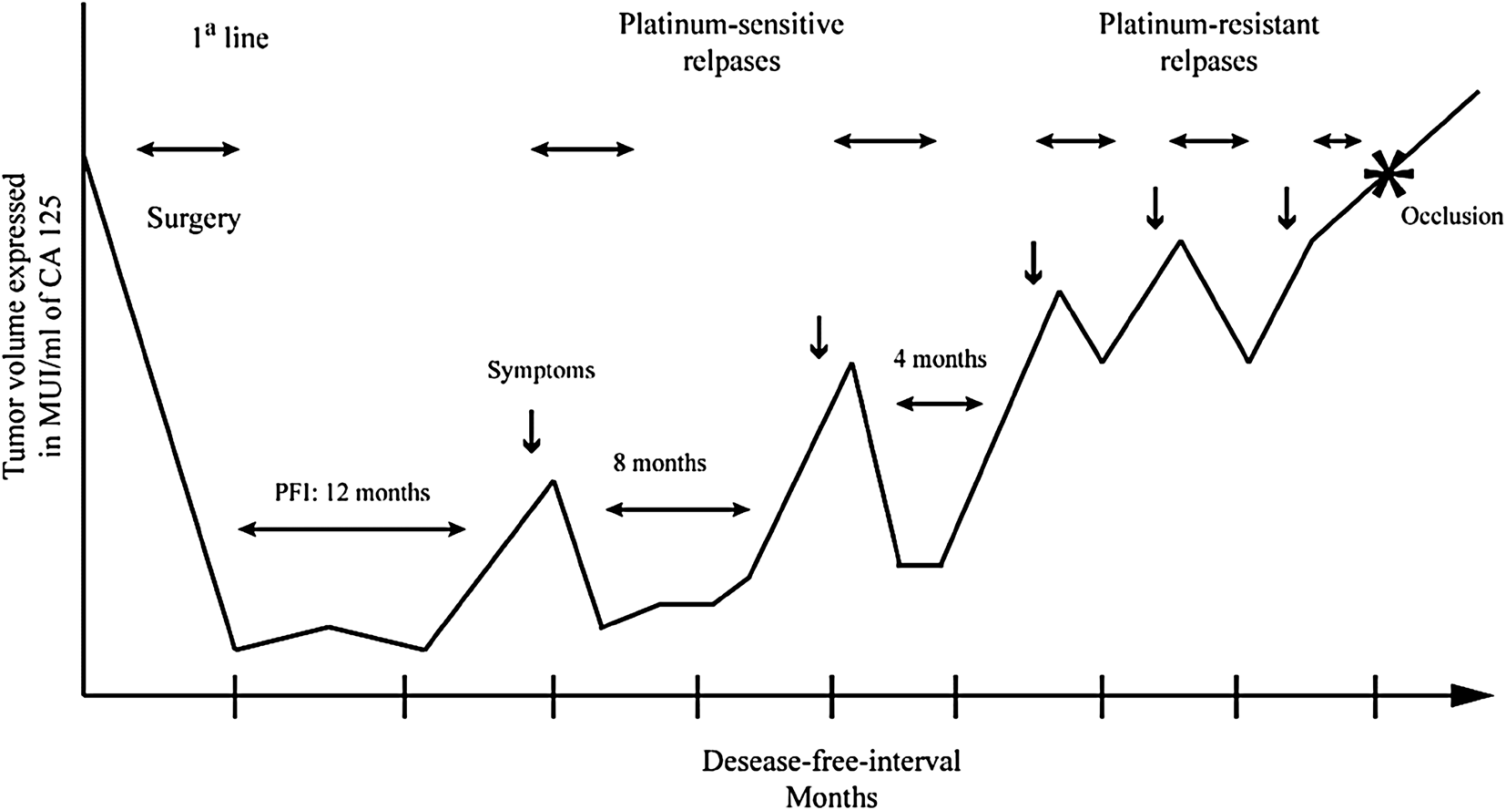
Natural history of ovarian cancer evolution

Ovarian cancer (OC) is the second most lethal gynecological neoplasia and the seventh cause of cancer-related mortality in women around the world (Globocan 2012). Nearly 70 % of the patients are diagnosed with advanced-stage due to the failure of screening methods for detecting early-stage disease (Partridge et al. 2009; Bast et al. 2007; Gohagan et al. 2000; Chudecka-Głaz 2015). Thus, most patients will relapse within the first 2 years after diagnosis, even after an optimal primary cytoreductive surgery and six cycles of the standard adjuvant chemotherapy with carboplatin/paclitaxel (Fig. 1) (International Collaborative Ovarian Neoplasm Group 2002).

Although several regimes have been evaluated to improve outcomes (Armstrong et al. 2006; Katsumata et al. 2013; Bookman et al. 2009; du Bois et al. 2014; Burger et al. 2011; Oza et al. 2015b), relapse seems unavoidable. The main objective of this review is to evaluate management of relapse and maintenance therapies in phase II/III trials. Therefore, I carried out a review based on the full-text published articles using key words “relapsed ovarian cancer” and “recurrent ovarian cancer” in all major medical article searchers.

### Biology of relapse

Metastases or relapses seem to be generated by “cancer stem cells” (CSCs) (Green 1989), a sub-population of cells, chemo and radio-resistant by expression of chemo resistant makers like aldehyde-dehydrogenase 1, efflux-drug transporters, or merely by staying quiescent in G0 phase of the cell-cycle, until a “driver event” (paracrine/endocrine factors) occurs and they proliferate to a more differentiated and chemo-sensitive population of cells (Charafe-Jauffret et al. 2009; Shah and Landen 2014; Tomao et al. 2014; Croker and Allan 2008; Zhang et al. 2014; O’Connor et al. 2014).

CSCs divide symmetrically (originate new CSCs) and asymmetrically (a daughter-cell starts to generate clones). So, CSCs originate relapse and guarantee their perpetuity and further relapses (Cojoc et al. 2015).

After chemotherapy (Ct) induced cytoreduction, the tumor microenvironment changes as it becomes less hypoxic. This “driver event” helps CSCs to proliferate, and its progeny does the same as they sense the new favorable conditions (Tomao et al. 2014); neo-vascularization(“angiogenic switch”) switches “on” and so this proliferating progeny becomes sensitive to chemotherapeutic drugs and PARPi; to repair DNA-damage that occurs during DNA-replication under hypoxia.

This is the biology underlying the maintenance phase after the cytoreduction achieved after second-line Ct for relapse; clinically manifested in complete or partial response.

### Management of relapse

There are several controversial issues (Walters Haygood et al. 2014):

*Early versus delayed treatment*, depending on the kind of “relapse”:

- an increase CA 125 with no other evidence of disease.
- or “clinical relapse”, evident through images (Ultrasound (US), Computed Tomography (CT) scan or PET/CT scan) or in physical examination.

MRC OVO5/EORTC 55955 included 1,442 patients, who after completion first line Ct were followed with CA 125 every 3 months (Rustin et al. 2010). Those who doubled baseline tumor marker in two consecutive measurements (n = 592) were randomized to an “early treatment” arm (N = 265). In the “delayed treatment” arm (264), the treatment was initiated upon clinical relapse.

With a median follow-up of 56.9 months (m), there was no benefit for the “early treatment” arm in overall survival (OS) (HR 0.98, IC 95 % 0.80–1.20, p = 0.85). Median survival (in months) in “early” versus “delayed” treatment: 25.7 m (IC 95 % 23.0–27.9) versus 27.1 m (IC 95 % 22.8–30.9).

The “early treatment” arm had a significantly shorter time to a second-line (4.8 m before (IC 95 % 3.6–5.3) and third-line of treatment. Quality of life (QoL) was deteriorated before (7.2 vs. 9.2 m with a good “Global health” score) and the time to deterioration was significantly longer in the “delayed treatment” arm (3.2 vs. 5.8 m, p = 0.002) (Rustin et al. 2010).

Therefore, CA 125 should be reconsidering in the follow-up of OC patients before starting and/or changing the treatment. Although it is useful for monitoring treatment efficacy, one should not modify the treatment, based *only* on this test.

*Surgery of relapse*. *Secondary cytoreduction* or *secondary debulking* is the surgery for resection of the site(s) of relapse to render the patient optimally debulked (R0) (Bristow et al. 2009).

Although most of the reports are retrospective series and subjected to “selection bias”, they report survival up to 35 m for patients who achieve an R0 after surgery of relapse (Bristow et al. 2009; Harter et al. 2006; Galaal et al. 2010; Wakabayashi et al. 2008). There is a score that predicts the chance of achieving an “optimal” secondary debulking (Harter et al. 2006; Salani et al. 2007). Globally, we can consider this intervention in “late” relapses (>12 m) or with a low volume of disease (Salani et al. 2007; Munkarah and Coleman 2004).

The results of AGO-DESKTOP (AGO Study Group 2015) and NCI (2007), evaluating the benefits of this surgery in relapses after 6 m of the last platinum-based Ct (platinum-sensitive relapse), are being awaited.

*Second-line chemotherapy* The combination is given for relapsed disease whether it is first, second or third line and depends mainly on the disease-free interval (DFI) (time between completion of first line Ct and clinical relapse; or progression-free interval (PFI) (time between the last Ct given for relapsed disease and progression).

According to these intervals, the relapse is:

#### Platinum-refractory/resistant

Relapses during platinum treatment (refractory) or with a disease-free interval (DFI)/PFI <6 months (resistant). Usually symptomatic with large-volume disease, these relapses are frequent later in the course of this disease (shorter PFI). Once the treatment has been chosen, one should not only consider overall response rate (ORR) to achieve symptomatic palliation (pain, ascites, etc.), but QoL improvement.

Many trials have compared single agent versus combination Ct in this setting, with no difference in ORR nor in progression free survival (PFS) for the combinations, which result in increased toxicity (Sehouli et al. 2008; Lortholary et al. 2012) Pegylated liposomal doxorubicin (PLD) has demonstrated similar ORR with a more favorable toxicity profile than with topotecan and gemcitabine (Gordon et al. 2004).

Although Ct combinations are not superior to single agent PLD, combination with bevacizumab (BEV) has improved the results (see “Bevacizumab”), and now is considered the best treatment in terms of ORR, PFS and QoL improvement in patients who had not received BEV previously (in combination with either weekly paclitaxel, PLD or topotecan).

#### Platinum-sensitive

Relapses occurring >12 m of last platinum-based Ct, usually with low-volume disease, eventually candidate for secondary cytoreduction.

At least, three phase III, randomized trials show benefit for platinum-combination Ct (plus PLD, gemcitabine or paclitaxel) versus platinum single-agent (Parmar et al. 2003; Sandercock et al. 2002; Pfisterer et al. 2006).

Given its low toxicity profile (particularly in elderly patients) and no cumulative neurotoxicity, the preferred regimen is carboplatin/PLD (Wagner et al. 2012; Kurtz et al. 2011; Brundage et al. 2012). Furthermore, BEV addition to platinum-based combinations (like carboplatin/gemcitabine) improves ORR and PFS results (Poveda et al. 2011, 2014; Aghajanian et al. 2015).

#### Partially sensitive to platinum

Disease-free survival (DFS)/PFS between 6 and 12 months from the last platinum-based Ct. Thus, artificially prolonging the platinum-free interval by incorporating a non-platinum regimen (trabectidin/PLD), saving platinum for a further relapse, has shown benefit in PFS and OS in the OVA 301 trial (Poveda et al. 2011). Although a similar proportion of patients in each arm of this trial received platinum-based Ct in subsequent relapse, the combination arm did so significantly later (Poveda et al. 2011).

INNOVATYON trial compares platinum-based combination versus trabectidin/PLD (followed by platinum-based Ct for further relapse) in this setting (DFI 6–12 months). The trial end points are DFS and OS. It has recently completed accrual and results are awaited (Poveda et al. 2014). Table 1 summarizes the Ct combinations/singe agent according to “platinum-free-interval”.

**Table 1:** Chemotherapy combinations/single agent according to platinum-free-internal

Platinum free interval	Sensibility to platinum	Regimen suggested
>12 m	Platinum-sensitive	Carboplatin/PLD Carboplatin/Paclitaxel Carboplatin/Gemcitabine
6–12 m	Platinum partially-sensitive	Carboplatin combination Trabecitidin/PLD
<6 m	Platinum resistant	PLD Weekly paclitaxel Topotecan Gemcitabine
Progression intra platinum treatment	Platinum-refractory	

### Targeted agents plus second-line chemotherapy

Anti-VEGF antibody (bevacizumab), VEGF dependent tirosine-kinase inhibitor (cediranib), and anti-angiopoietin 1–2 pepto-antibody (trebananib) are targeted agents evaluated in clinical trials in association with chemotherapy in different settings of relapsed disease.

#### Bevacizumab

Based on the results obtained by adding bevacizumab to first-line chemotherapy in GOG 218 and ICON 7 trials (Burger et al. 2011; Oza et al. 2015b) (benefit in DFS), BEV was also evaluated in two phase III, randomized trials in the relapse setting: OCEANS (Aghajanian et al. 2015) and AURELIA (Pujade-Lauraine et al. 2014; Poveda et al. 2015).

OCEANS randomized patients with platinum-sensitive relapse to chemotherapy [carboplatin/gemcitabine (GC)] + placebo (PL) (GC + PL) versus the same regimen + bevacizumab (GC + BEV) concomitant with chemotherapy and as maintenance until disease progression. The GC + BEV arm showed benefit in ORR (78.5 vs. 57.4 % p < 0.0001) and PFS (8.4 vs. 12.4 m (HR 0.48, p < 0.0001); although no difference was observed in OS (GC + BEV: 33.6 m; GC + PL: 32.9 m; hazard ratio = 0.95; log-rank p = 0.65) (Aghajanian et al. 2015).

In the platinum-resistant setting, the AURELIA trial also randomized patients to a Ct + PL arm (investigator’s choice: weekly paclitaxel, PLD or topotecan) versus the same agents + BEV, followed by a maintenance phase until disease progression. As shown in OCEANS, the combination with BEV arm was significantly superior in ORR (12.6 vs. 30.9 %, considering response by CA 125 and RECIST) and PFS (3.4 vs. 6.7 m (HR0.48, p < 0.0001), albeit no difference in OS between the arms (HR 0.85 p NS) was observed. Although the study was not designed to detect differences between the three Ct + BEV combinations, the weekly paclitaxel (Pcl) + BEV performed better in ORR (ORR CT + BEV: weekly Pcl 23.5 %, PLD: 10.4 % and topotecan: 19.5 %) (Poveda et al. 2015).

Patients receiving BEV improved global OC QoL scores, and had a significant reduction in the number of paracentesis needed to alleviate ascites (Stockler et al. 2014).

Table 2 compares the results of the four phase III trials evaluating BEV in OC either in first line setting or at relapse (GOG 218, ICON 7, OCEANS y AURELIA), as well as the sub-group analysis of the “high-risk” group in the ICON 7 (Stage III R1 and IV) (Burger et al. 2011; Oza et al. 2015b; Pujade-Lauraine et al. 2014; Poveda et al. 2015; Ledermann et al. 2013; Raja et al. 2011; du Bois et al. 2016; European Medicine Agencies 2016; Matulonis et al. 2016; Marchetti et al. 2015; Lopez et al. 2013).

**Table 2:** Phase II/III trials with targeted agents in ovarian cancer (du Bois et al. 2014, 2016; Burger et al. 2011; Oza et al. 2015a; Aghajanian et al. 2015; Poveda et al. 2015; Ledermann et al. 2013; Matulonis et al. 2016; European Medicine Agencies 2016; Marchetti et al. 2015)

First line					Relapse				
					Platinum sensitive (PFS > 6 m)			(PFS 0–12 m)	Platinum resistant (<6 m)
	GOG 218 Bev (11)	ICON7 Bev (12)	AGO Pazopanib (10)	AGO Nintendanib (48)	OCEANS Bev (42)	ICON6 Ced (46)	STUDY 19 Olap (50)	Trinova –1 Trebananib (52)	AURELIA Bev (44)
DFS/PFS*	3.8	1.7	5.6	0.7	4.0	3.1	4.0	1.8	3.3
DFS/PFS HR	0.72	0.81	0.77	0.84	0.48	0.57	0.35 BRCA^m^ 0.18	0.66	0.48
OS*	0.4	0.9	NA	NR	−1.8	2.7	2.0	1.7	3.3
OS HR		0.91 (NS)	0.99 (NS) (final)	NR	1.03 (NS)	0.70	0.88 (NS)	0.86 (NS)	0.85 (NS) (final)

* Difference in months

Given the higher HR in the trials (GOG 218, ICON 7, OCEANS and AURELIA trials) evaluating BEV in the relapse setting, we can infer that patients with large-volume disease (platinum-resistant relapse) obtain the greatest reduction in risk of progression and a greater benefit in ORR and QoL.

FDA and EMA guidelines only approve BEV for relapsed patients who have not received this agent in the first line. Moreover, for platinum-sensitive relapse, the approval is only for “first” relapse.

#### Cediranib

It inhibits vascular endothelial growth factor (VEGF) receptor 1, 2 and 3 tyrosine kinases. ICON 6 was a phase III, randomized trial which evaluated the combination of cediranib (Ced) with platinum-based Ct, followed by maintenance therapy for platinum-sensitive relapsed (Monk et al. 2015); patients were randomized (n = 465) to Ct + Ced (20 mg P.O daily) versus placebo. The experimental arm showed a significant difference of 3.2 months in PFS and 2.7 m in OS (HR 0.68; log-rank test p = 0.0022). Most frequent adverse events were hypertension, diarrhea, hypothyroidism, dysphonia, bleeding, proteinuria, and fatigue (Raja et al. 2011). Despite the benefit in PFS and OS, the submission for approval of this drug by FDA and EMA was withdrawn (Lord and Ashworth 2012).

Cediranib + olaparib (PARPi) combination has shown activity as a non-chemotherapy treatment for platinum-sensitive relapsed OC (Lord and Ashworth 2012). It will be compared in a three-arm, phase III trial versus olaparib versus standard platinum-based Ct in platinum-sensitive relapse (Raja et al. 2011; Monk et al. 2015; Lord and Ashworth 2012).

#### Trebananib

It is a peptidic antibody (AMG 386) that blocks interaction between angiopoietin receptors Ang 1 (promotes good quality neo vessels growth) and Ang 2 (related to the number of neo vessels), and their ligand Tie 2. Both receptors are over-expressed in OC.

Trebananib has shown activity as single agent in phase I trials and associated to weekly Pcl in phase II trials, showing benefit in PFS + Ct (Lord and Ashworth 2012).

TRINOVA 1 included 919 heavily pre-treated patients (≥3 lines) with relapsed OC and a platinum-free interval ≤12 m (nearly 50 % of patients in each arm were “platinum- resistant”). They were randomized to trebananib 15 mg/kg/week + Pcl 80 mg/m^2^/week versus the same chemotherapy regimen + placebo. Median follow-up: 18 m; primary end-point was PFS. The experimental arm had a significant advantage in PFS (HR 0.66 (IC 95 % 0.57–0.77) p < 0.001). Although all patients showed improvement in this end-point, the sub-group with ascites benefited the most (HR 0.72 (IC 95 % 0.85–0.93) p < 0.011). Trebananib arm also had better ORR and a longer time to subsequent treatment. Non-significant differences were observed in OS (HR 0.95 (IC 95 % 0.81–1.11)).

Although the difference in Grade 3–4 serious adverse events was non-significant, the experimental arm showed a higher incidence of localized edema, pleural effusion, and ascites. This was the most frequent cause of treatment discontinuation (20 % of the patients) (Monk et al. 2015).

#### PARP inhibitors

These agents impair proper DNA repair by inhibiting PARP (Poly (ADP-ribose) polymerase) that has a key role in “base excision repair (BER), through which single strand DNA damage is repaired. DNA poly adenosil ribosilation (PAR) is a key pathway where gathering all the machinery needed for reparation where single strand DNA is damaged (Marchetti et al. 2015; Lord and Ashworth 2012; Li and Yu 2015; Mukhopadhyay et al. 2011, Chionh et al. 2011; Schreiber et al. 2006).

By inhibiting PARP, single strand DNA remains unrepaired and the “replication fork” (DNA polymerase complex) is stalked, hence single strand breaks turn into a double strand-break. BRCA 1 & 2 proteins work as a scaffold for other factors important in “homologous recombination” (Hr), a high fidelity process to repair this type of DNA damage. Hr occurs only in G2 or M phases of the cell cycle because it requires the presence of the sister chromatid as a template to create an exact copy of the impaired DNA fragment.

Malfunction of these proteins—by germinal or somatic mutation or epigenetic inactivation of the genes (methylation)—creates a state of homologous-recombination efficiency (HRd), and the cell recurs to less efficient (low fidelity) mechanisms to repair DNA, like non-homologous end joining (NHEJ), through which the damaged fragment is excised and the 3′ and 5′ ends are joined. This implies cell loss of variable-length portions of DNA, which eventually leads to an extensive genomic loss and the cell incapability to survive (Marchetti et al. 2015; Schreiber et al. 2006).

In HRd (like BRCA 1 & 2 mutated) cells, by inhibiting PARP, we can create a “synthetic lethality” state: single-strand DNA damage cannot be repaired by BER and the cell is forced to use an inefficient mechanism to repair double-strand breaks, like NHEJ. This results in a large DNA loss. Without it, the cell losses critical genes for surviving and finally the cell dies (Audeh et al. 2010; Chen et al. 2013; Cancer Genome Atlas Research Network 2011; Liu et al. 2014b; Fong et al. 2010).

Around 20 % of OC patients have a germinal or somatic BRCA 1 or 2 mutated (BRCA^m^) plus 11 % with epigenetic inactivation of these genes (Oza et al. 2015a). Considering mutations in other genes involved in HR, up to 50 % of OC patients have a HRd, the so-called “BRCA-ness syndrome”. This phenotype is more frequent (but not exclusive) in “platinum-sensitive relapse” OC. Thus, failure to repair the damage caused in DNA by platinum (inter/intra strand adducts that would normally be repaired by Hr or BER) is a surrogate of an HRd (Oza et al. 2015a; Ledermann et al. 2014).

Different PARP inhibitors (PARPi) have been or are under clinical investigation in different OC settings:

1. associated to first-line chemotherapy and as maintenance (veliparib),
2. associated to second-line chemotherapy and as maintenance after platinum-sensitive relapse (olaparib, rucaparib, niraparib)
3. as single agent in heavily pre-treated patients (olaparib) (Cancer Genome Atlas Research Network 2011).

### Trials with PARPi

These trials evaluate PARPi (olaparib) associated with second-line Ct and, as maintenance treatment or as maintenance only.

An open label phase II trial randomized 162 platinum-sensitive relapsed OC patients to platinum-based Ct (carboplatin-paclitaxel) + olaparib 200 mg P.O BID (days 1–10) concurrent with chemotherapy (81 patients) or chemotherapy alone (75 patients); 121 patients continued receiving olaparib as maintenance (400 mg P.O, BID, until progression) or placebo (66 in the olaparib + chemotherapy arm and 55 in the chemotherapy alone arm) (Gourley et al. 2014).

BRCA mutation status was known in 107 patients: 41 (38 %) had BRCA mutated (20 in the experimental arm and 21 in the Ct alone arm). PFS was significantly longer in the olaparib + Ct arm (median 12.2 m [95 % CI 9.7–15.0]) than in the Ct alone arm (median 9.6 m [95 % CI 9.1–9.7) (HR 0.51 [95 % CI 0.34–0.77]; p = 0.0012), particularly in BRCA^mut^ (HR 0.21 [0.08–0.55]; p = 0.0015). In the concurrent phase, the most frequent adverse events with a difference of at least 10 % between the arms were alopecia (60 [74 %] out of 81 vs. 44 [59 %] out of 75), nausea (56 [69 %] vs. 43 [57 %]), neutropenia (40 [49 %] vs. 29 [39 %]), diarrhea (34 [42 %] vs. 20 [27 %]), headache (27 [33 %] vs. 7 [9 %]), peripheral neuropathy (25 [31 %] vs. 14 [19 %]), and dyspepsia (21 [26 %] vs. 9 [12 %]); most of them were mild to moderate. Grade ≥3 events were neutropenia (in 35 [43 %] out of 81 patients in the experimental arm vs. 26 [35 %] out of 75, and anemia (7 [9 %] vs. 5 [7 %]). Serious adverse events were reported in 12 (15 %) out of 81 patient in the olaparib + Ct arm and in 16 out of 75 (21 %) in the standard arm (Gourley et al. 2014).

Although it was not designed to evaluate the benefit of adding olaparib to Ct or as maintenance only; lack of difference between the arms in ORR and the split of the PFS curves at the beginning of the maintenance phase suggest that the benefit of adding PARPi occurs in this period. Toxicities were more conspicuous in the concurrent phase, and affected tissues, where the impact of incorporating an agent affecting DNA repair would be greater: bone marrow and gastro-intestinal mucosae.

### Maintenance treatment after relapse

This treatment is given after partial or complete response after second-line Ct (±secondary cytoreductive surgery) for relapsed OC until progression; thus it should be:

- effective to achieve a longer time to progression (PFS)
- tolerable as a long-lasting treatment (i.e. ≥2 years), without impairing QoL.

The same principles apply to the biological mechanisms underlying of this maintenance phase. So, the targetable events are angiogenesis and DNA replication/repair.

### Maintenance with anti-angiogenic agents

#### Bevacizumab

OCEANS (Aghajanian et al. 2015) and AURELIA (Pujade-Lauraine et al. 2014) evaluated BEV as maintenance either after platinum-sensitive or platinum-resistant relapse. Adding bevacizumab to OC treatment seems to be directly proportional to the volume of disease, given that the greatest benefit in reducing the risk of progression was seen in patients with the greatest volume of disease at relapse (platinum-resistant), who also obtained a greater advantage in ORR and in QoL (Pujade-Lauraine et al. 2014; Poveda et al. 2015).

In both trials, BEV was well tolerated, had low-discontinuation rates and less than 5 % Gr 3–4 adverse events, mostly hypertension, arterial/venous thromboembolic events or fistulae. At progression, roughly 30 % of patients in the placebo arm received an anti-angiogenic agent, which may explain the lack of OS difference between the arms.

At relapse, bevacizumab has only been approved for patients who had not received it in first line.

#### Cediranib

See “Cediranib” in “Targeted agents plus second-line chemotherapy” section.

### Maintenance with PARPi

Olaparib was evaluated as maintenance treatment after platinum-sensitive relapse in “Study 19” (Matulonis et al. 2016). It included 265 patients regardless their BRCA status who after achieving complete or partial response to induction platinum-based Ct were randomized to olaparib 400 mg P.O, BID, until 1progression or PL.

Olaparib arm showed a significantly longer PFS (8.4 vs. 4.8 m (HR 0.35 [95 % CI 0.25–0.49] p < 0.001), time to first subsequent treatment and time to subsequent relapse. However, there were no differences in OS: HR 0.94 [95 % CI 0.63–1.39] p = 0.75. It was generally well tolerated. The most common toxicities were hematological (anemia and leucopenia), gastro-intestinal (nausea, vomiting, and abdominal pain), and others like asthenia and fatigue. Most of the toxicities were Grade 1–2 and could be managed with dose reduction. The risk of myelodysplastic syndrome and acute leukemia was <3 %. By January 2014, 19 patients in the olaparib arm continued under treatment, 24 (18 %) out of 136 patients in the experimental arm had received olaparib for more than 3 years.

When the study began, BRCA status was known only for 30 % of patients, but a subsequent analysis was performed to evaluate the differential benefit of BRCA^m^ patients. As expected BRCA 1 & 2^m^ patients (56 % in the olaparib arm vs. 50 % in the PL arm) had a greater benefit in all end-points: median PFS of 11.2 m ([95 % CI 8.3–no calculable] vs. 4.3 m [3.0–5.4]; HR 0.18 [0.10–0.31]; p < 0.0001).

**Fig. 2:**
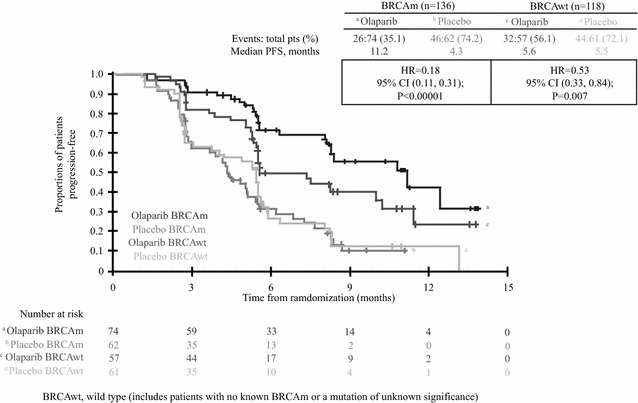
“Study 19”, progression free survival according to BRCA status. Adapted from Matulonis et al. (2016), Ledermann et al. (2014), Birrer et al. (2015)

This is the greatest benefit obtained by any agent in terms of PFS in OC. However, BRCA^wt^ patients also benefited from olaparib maintenance, although the difference was less (7.4 m [5.5–10.3] vs. 5.5 m [3.7–5.6]; HR 0.54 [0.34–0.85]; p = 0.0075) (Fig. 2). Since up to 50 % of platinum-sensitive relapsed patients have HRd, this would make them sensitive to this PARPi. Nevertheless, based on the greater benefit observed in BRCA^mut^ (germinal or somatic), EMA approved olaparib as maintenance therapy after response to platinum-based Ct in this population at ≥1st platinum-sensitive relapse.

Grade ≥3 toxicities in the olaparib arm were fatigue (in 10 [7 %] patients vs. 4 [3 %] in the placebo arm) and anemia (7 [5 %] vs. 1 [<1 %]). Serious adverse events were reported in 25 patients (18 %) in the experimental arm and 11 (9 %) in the PL. There were no differences in tolerability between BRCA^mut^ and BRCA^wt^ population (Marchetti et al. 2015).

In a second interim analysis (with 38 % of maturity) OS was not significantly different between the two groups (HR 0.88 [95 % CI 0.64–1.21]; p = 0.44); the same applies to BRCA^mut^ patients (HR 0.73 [0.45–1.17]; p = 0.19) and BRCA^wt^ (HR 0.99 [0.63–1.55]; p = 0.96).

### Predictive factors in OC

Although adding targeted agents to Ct or as maintenance has improved the results obtained with Ct alone (longer PFS and higher ORR), it is necessary to identify “predictive factors” to distinguish which subgroup of patients benefit the most.

#### Bevacizumab

ICON 7 (n = 284 total, 18.6 % patients analyzed) evaluated if the differentiated, immuno-reactive, mesenchymal, and proliferative genomic profiles described in OC^65^ derived differential benefit from BEV (Gourley et al. 2014; McNeish et al. 2015).

The mesenchymal and proliferative sub-types were benefited by adding BEV to first-line Ct and the immuno-reactive did not (Birrer et al. 2015). A validation analysis in a prospective larger group of patients is required to confirm these data, as well as to assess if this differential benefit is applicable to relapsed patients.

At ASCO 2015, data were presented showing the level of expression of CD 31 as a predictive factor for BEV (Liu et al. 2014a).

#### PARPi

As seen before, BRCA mutational status is not only a predictive factor for response to Ct (Alsop et al. 2012) (platinum combinations, tarbectidin/PLD, PLD), but also identifies patients who benefit the most with olaparib maintenance therapy (Alsop et al. 2012). Since up to 30 % of BRCA^mut^ do not have family history of cancer and 25 % are older than 65 years old (SGO 2014), the Society of Gynecological Oncology recommends to test all patients with OC at diagnosis and to perform a complete mutational analysis of these genes through NGS (next generation sequence).

BRCA 1 & 2 are not the only genes responsible for a HRd. So, different panels of genes are being tested spanning between 5 and 25 genes involved in Hr, trying to enlarge the spectrum of patients potentially sensitive to PARPi.

ARIEL 2 validated a biomarker assay which could predict benefit with rucaparib as maintenance therapy after platinum-sensitive relapse (McNeish et al. 2015; Liu et al. 2014a). Through whole genomic sequencing from the tumor, a genomic signature called “LOH (loss of heterocigozity) signature” was identified, which is the expression of the large un-replicated areas of DNA, shown in the genomic sequencing as “DNA scars”. Patients with this “LOH signature” (“biomarker positive”, similar to the one BRCA^m^ patients had), benefited the most with this PARPi not only in ORR, but also in PFS in comparison with the ones not expressing this signature (“biomarker negative”), and behave like BRCA^m^ patients (n = 25) [HR 0.61(IC 95 % 0.41–0.92)] (Alsop et al. 2012).

### Non-chemotherapy combinations at relapse

Olaparib (Olap) and anti-angiogenic Ced combination was compared against Olap single agent in platinum-sensitive relapsed ovarian cancer. Ninety patients were randomized to Olap 400 mg BID P.O (46 patients) or the combination of Ced/Olap (Olap 200 mg BID; Ced 30 mg P.O daily) (44 patients). The combination arm showed benefit in ORR and PFS especially in BRCA^wt^ patients; 48 patients were BRCA^mut^ (25 Olap; 23 Ced/Olap). Median PFS was 9.0 m in the Olap arm and 17.7 m for Ced/Olap (HR 2.9, 95 % CI 1.5–5.6, p = 0.001). For ORR: Olap 56 % and 84 % for the combination (p = 0.008). Gr 3/4 toxicities were 70 % in the Ced/Olap arm and 7 % for Olap. The most frequent were fatigue (27 % Ced/Olap vs. 7 % Olap), diarrhea (23 vs. 0 %), and hypertension (39 vs. 0 %).

**Fig. 3:**
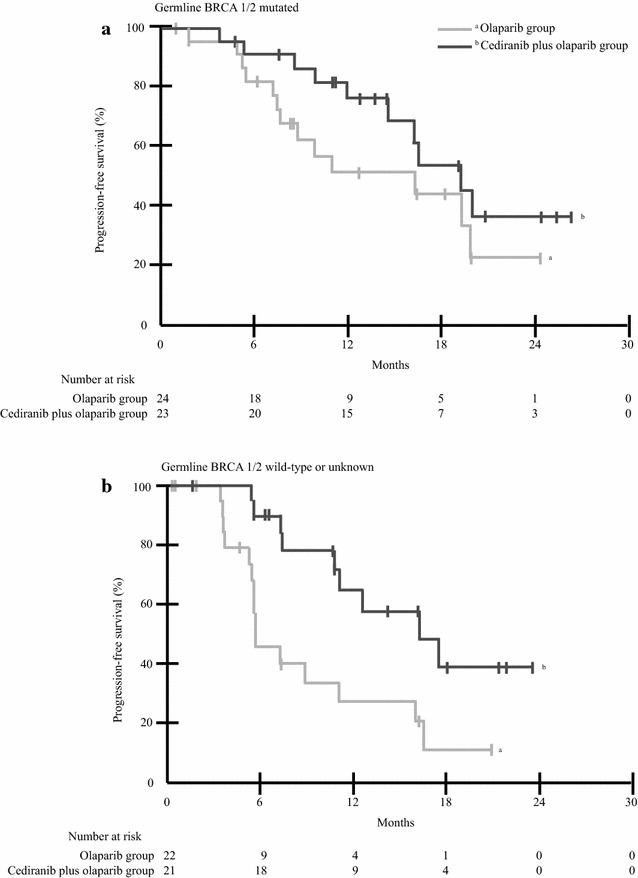
Ceridanib + olaparib versus olaparib in platinum sensitive relapse (PFS according to BRCA status). Adapted from Liu et al. (2014a)

The differential benefit for BRCA^wt^ patients may be explained by the generation of oxygen reactive species by Ced, which damage DNA pushing the cell to use a repairing system inhibited by the PARPi (Liu et al. 2014b; SGO 2014; Goss et al. 2009; NCI 2015) (Fig. 3).

This combination is being tested against chemotherapy in platinum-sensitive relapsed OC (du Bois et al. 2014).

## Conclusions

Management of relapsed OC involves many decisions; thus, there is still no firm clinical evidence: (1) early (based only on a raising CA 125) versus delayed treatment of relapse; (2) to perform or not secondary cytoreductive surgery; (3) what chemotherapy combination should be used (platinum vs. non-platinum); (4) anti-angiogenic agents in the first line versus at relapse, and (5) how to select the population who may benefit with PARPi maintenance therapy.

A deeper knowledge of the biology of the maintenance phase, as well as the molecular and cellular events (where cancer stem cells would play an important role in relapse/progression) may lead to targeted therapeutic strategies which would replace chemotherapy combinations for non-chemotherapy regimens, based on patient’s specific predictive factors.

So far, BRCA mutational status is the only predictive factor in OC, and the benefit obtained with PARPi (Olap) in BRCA^m^ has been the greatest among the different agents used in this disease. The reason is that for the first time in OC, a treatment is used based on a predictive factor.

The challenge is to identify other genes or a genomic signature that could enrich the population who may benefit from these agents, as well as other predictive factors for antiangiogenic agents.

This may allow tailoring specific therapies to obtain the greatest benefit with low-toxicity profile in a disease in which maintenance therapies delay progression/relapse.
